# Editorial: Exploring mental health in vulnerable populations in developing countries

**DOI:** 10.3389/fpubh.2025.1664925

**Published:** 2025-07-31

**Authors:** Jacob Owusu Sarfo

**Affiliations:** Department of Health, Physical Education and Recreation, University of Cape Coast, Cape Coast, Ghana

**Keywords:** mental health, low- and lower-middle-income countries, vulnerable populations, developing countries, mental illness, health system

Globally, mental health is increasingly recognized as a global health priority, although there exist significant disparities, especially in low- and middle-income countries (LMICs). According to the World Health Organization, Mental health is “*a state of mental wellbeing that enables people to cope with the stresses of life, realize their abilities, learn well and work well, and contribute to their community*” (1). Notwithstanding the growing evidence of mental health issues in developing countries, a research gap still exists on the holistic measurement and description of the problem, including interventions, policies, actions, and programs to mitigate mental health challenges.

In low-resource regions, vulnerable populations are often exposed to intersecting risks in the form of trauma, poverty, gender-based violence, under-resourced healthcare systems, and conflict. Together, these factors contribute to the burden of mental health disorders and limit opportunities for effective prevention and care. Our Research Topic brings together diverse empirical contributions, especially from Asia and Africa, which explored mental health outcomes, risk factors, and intervention pathways in vulnerable populations. From trauma-informed care to aging, and maternal health to structural violence, the included studies reflect the growing need to critically examine mental health not in isolation, but as embedded within broader social, cultural, geopolitical, and economic systems.

One of the essential topics evident in this Research Topic is the nature of trauma, discrimination, and internalized harm. Trauma, as a mental health concept, can shape psychological functioning well into adulthood, primarily when it occurs in early life. Wang and Wang investigated adult survivors of childhood sexual abuse in China, highlighting the mediating roles of rumination and perceived discrimination in sleep disturbance. Their study underlines the importance of trauma-informed approaches and the compounding effects of stigma on psychological and physiological health outcomes (1) Similarly, Ahmead et al. turned attention to healthcare providers working in conflict-affected Palestine. Their study revealed alarming levels of post-traumatic stress disorder among mental health professionals themselves, advocating for the integration of psychological support and supervision systems for frontline workers (Ahmead et al.).

Another key theme highlighted in the Research Topic is maternal and reproductive mental health. For instance, Chala et al. carried out a comparative study on antenatal depression among women in urban and rural areas of Ethiopia, revealing notable differences in both prevalence and contributing factors based on location. This work emphasizes the need for tailored mental health services in maternal healthcare, particularly in rural areas where access is limited. Also, it shows the importance of location-specific interventions and support systems in promoting the mental health of vulnerable populations in low-resourced settings.

Aside from mental health cases like depression, Gedefa et al. conducted a study in Gambella, Ethiopia, highlighting the widespread occurrence of intimate partner violence and its strong association with significant psychological distress. Their findings underscore how gender-based violence must be treated as both a public health and a mental health crisis, requiring coordinated community and health system responses (Gedefa et al.). Moreover, it points to an urgent need for culturally sensitive mental health care integrated into response systems in regions with high rates of intimate partner violence.

Addressing mental health within disability, inferiority, and social support context as a theme featured some essential articles. For instance, a study by Liu et al. surveyed individuals with physical disabilities in China and found that nearly half suffered from anxiety and over 60% from depression and feelings of inferiority. The study revealed that people who felt they had more social support tended to feel less inferior, highlighting the internal psychological burden of structural stigma (Liu et al.). These findings emphasize the importance of empowerment-based interventions that enhance perceived value and community inclusion among individuals with disabilities.

Another thematic area of relevance in the Research Topic is conflict, displacement, and student mental health. Ahmead et al. investigated the psychological impact of the October 2023 conflict on Palestinian university students, indicating widespread symptoms of depression and anxiety. Vulnerable students reported using both adaptive and maladaptive coping mechanisms, underscoring the need for institutional mental health support within academic settings in conflict zones (Ahmead et al.).

Besides, aging, cognition, and intergenerational ties as a sub-theme is captured by a study by Hu et al.. The study observed that the challenges of aging populations in LMICs are increasingly mental as well as physical. Using a longitudinal data, the study demonstrates that communication with children had a protective effect on cognitive aging among middle-aged and older adults. This reinforces the relevance of familial relationships as a valuable intervention for mental health promotion in vulnerable aging populations.

Finally, the Research Topic captured health systems, policy, and community mental health interventions within vulnerable populations. For instance, to enhance the screening of post-traumatic stress disorder (PTSD), Meffert et al. validated a two-item PTSD screener for East Africa. The tool showed strong performance relative to more complex measures, indicating the necessity and utility of ultra-brief instruments for scalable screening in resource-constrained healthcare systems. Additionally, Dan-Ni et al. examined the prevalence and risk factors associated with anxiety and depression among patients with multi-drug or rifampicin-resistant tuberculosis (MDR/RR-TB) in southern China. Their findings identified that individuals with MDR/RR-TB had an elevated risk of anxiety and depression, and highlight the need to assess and manage psychological distress during care.

Overall, this Research Topic offers critical insights into the mental health needs of vulnerable populations in LMICs. This Research Topic has implications for future policy, systems-level change, training of mental health workers, funding, and research.
